# Conspecific Plant-Soil Feedbacks of Temperate Tree Species in the Southern Appalachians, USA

**DOI:** 10.1371/journal.pone.0040680

**Published:** 2012-07-10

**Authors:** Kurt O. Reinhart, Daniel Johnson, Keith Clay

**Affiliations:** 1 Fort Keogh Livestock and Range Research Laboratory, United States Department of Agriculture – Agricultural Research Service (USDA-ARS) Miles City, Montana, United States of America; 2 Department of Biology, Indiana University, Bloomington, Indiana, United States of America; DOE Pacific Northwest National Laboratory, United States of America

## Abstract

Many tree species have seedling recruitment patterns suggesting that they are affected by non-competitive distance-dependent sources of mortality. We conducted an experiment, with landscape-level replication, to identify cases of negative distance-dependent effects and whether variation in these effects corresponded with tree recruitment patterns in the southern Appalachian Mountains region. Specifically, soil was collected from 14 sites and used as inocula in a 62 day growth chamber experiment determining whether tree seedling growth was less when interacting with soil from conspecific (like) than heterospecific (other) tree species. Tests were performed on six tree species. Three of the tree species had been previously described as having greater recruitment around conspecifics (i.e. facilitator species group) compared to the other half (i.e. inhibitor species group). We were then able to determine whether variation in negative distance-dependent effects corresponded with recruitment patterns in the field. Across the six species, none were negatively affected by soil inocula from conspecific relative to heterospecific sources. Most species (four of six) were unaffected by soil source. Two species (*Prunus serotina* and *Tsuga canadensis*) had enhanced growth in pots inoculated with soil from conspecific trees vs. heterospecifics. Species varied in their susceptibility to soil pathogens, but trends across all species revealed that species classified as inhibitors were not more negatively affected by conspecific than heterospecific soil inocula or more susceptible to pathogenic effects than facilitators. Although plant-soil biota interactions may be important for individual species and sites, it may be difficult to scale these interactions over space or levels of ecological organization. Generalizing the importance of plant-soil feedbacks or other factors across regional scales may be especially problematic for hyperdiverse temperate forests where interactions may be spatially variable.

## Introduction

Cryptic soil biota may associate with and affect tree abundance, habitat association, and the diversity of entire forests [1]–[6]. Two studies have correlated either tree relative abundance [1] or tree seedling recruitment patterns [7] with soil biota effects/feedbacks. Soil-borne pathogens may cause these effects but their effects are not always uniform across species and sites and may limit how they are generalized [8], [9]. Some studies suggest a degree of host-specificity by soil-borne pathogens producing disease dynamics that are distance-, density-, and/or frequency-dependent [3]–[5], [9], [10]. These results support the Janzen-Connell Hypothesis, which predicts that host-specific enemies reduce the survivorship of offspring that establish close to parents or when offspring are abundant [11], [12]. In these cases, the pathogens appear to track the distribution and abundance of suitable hosts [9].

Disease dynamics may also be affected by habitat characteristics (e.g. gaps vs. forest understories) and variation in susceptibility among potential host species [2], [6], [13], [14]. For example, some studies indicate that pathogenic activity is less in open habitats (e.g. forest gaps) than in closed canopy forests [2], [13], [15] and that shade tolerant species are more tolerant to disease than shade-intolerant species [6], [15] but see [16]. However, others have proposed that species with small seed sizes are most susceptible to natural enemies [17]. These different bodies of research suggest the expression of disease is determined by a three-way interaction among characteristics of the pathogen (e.g. virulence and abundance), the host (e.g. factors relating to susceptibility), and the environment (e.g. conditions favoring disease) [18]. These sources of variability motivated this study comparing plant-soil biota interactions across several tree species and looking for general patterns at a regional scale instead of focusing on interactions at a local scale which most studies have done. Here our goal was to determine if plant-soil biota interactions are a regional driver of recruitment patterns in temperate forests of the southern Appalachian Mountains.

To accomplish this, we selected multiple species known to differ according to a published *Inhibition* index that ranked the recruitment patterns of tree species based on forest inventory data of plots throughout the region [19]. This index characterizes recruitment of seedlings and saplings around conspecific trees versus recruitment in areas without conspecific trees [19]. This was useful because species with most of their recruits occurring in plots without conspecific trees were considered more likely to be affected by non-competitive distance-, density-, or frequency-dependent sources of mortality. Greater susceptibility to disease, especially those caused by host-specific pathogens, would favor dispersal to areas with low pathogen loads. After detecting variability in recruitment patterns among tree species with a regional dataset [19], our goal was to relate these patterns to regional processes and focused on the importance of negative soil biota effects. We selected an equal number of species from the two extremes of this classification which we refer to as inhibitors (i.e. rarely recruit near conspecifics) or facilitators (i.e. commonly recruit near conspecifics). The selected species were then used in a regional soil biota experiment that tested three hypotheses. H1) Overall seedling growth and survival will be less when inoculated with soil from conspecific adult trees than heterospecifics, consistent with predictions for the Janzen-Connell Hypothesis. H2) Species classified *a priori* as inhibitors will be more negatively affected by soil inocula from conspecifics relative to heterospecifics than species classified as facilitator species indicating their greater susceptibility to soil-borne pathogens. H3) Overall seedling survival will be greater for species classified as facilitators than those classified as inhibitors. Addressing these hypotheses will determine whether plant-soil biota interactions are a general mechanism explaining the variation in cohorts of species with different recruitment patterns at a regional scale.

## Results

### Soil Source Experiment

**Figure 1:**
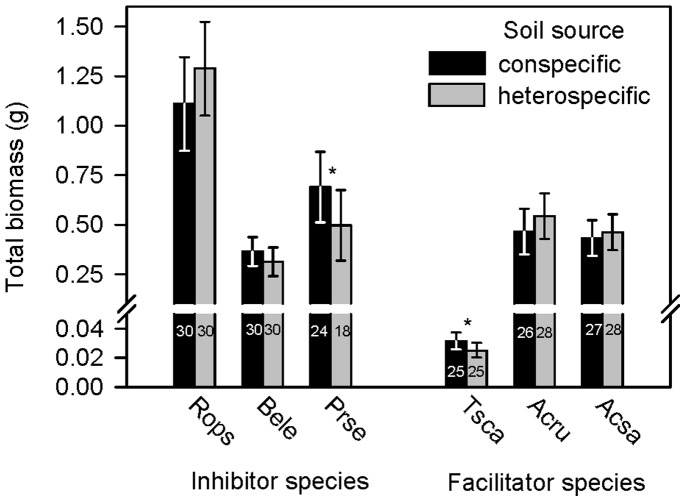
Effect of soil source (conspecific vs. heterospecific) on the least squares mean (LSM) estimates of seedling total biomass and 95% CI. Numbers inside of bars indicate the number of seedlings out of 30 that were alive at 62 days. Species are grouped by their *Inhibition* index. Species names and statistical results reported in Table 1. *P<0.10.

Overall, species did not grow more in soil collected near heterospecific trees than conspecifics (H1) (Fig. 1). Furthermore, there was a marginally significant negative effect of soil from heterospecifics vs. conspecifics on the survival of *Prunus serotina* seedlings (GLMM, F_1,53_ = 2.77, *P* = 0.10; Fig. 1), and other species were unaffected (H1)(*P*≥0.40). Contrary to H1, one inhibitor (*Prunus serotina*) and one facilitator species (*Tsuga canadensis*) had greater total biomass production in pots inoculated with soil from conspecifics than from heterospecifics (Fig. 1, Table 1). Although we anticipated inocula from conspecifics to have more negative effects on growth and survival than inocula from heterospecifics for all species, we predicted the magnitude of this variation would be greatest for species classified as inhibitors (H2). Contrary to this prediction, there were no general differences in responsiveness to inocula for species classified as inhibitor vs. facilitator species (H2)(Fig. 1, Table 1).

**Table 1:** Mesic deciduous forest sites containing inhibitor, facilitator, and other tree species (i.e. *Quercus rubra*).

				inhibitor				facilitator				
Area	Site	Lat/Long	Elev. (m)	*Betula lenta*	*Prunus serotina*	*Quercus* *coccinea*	*Robinia pseudoacacia*	*Acer rubrum*	*Acer saccharum*	*Fagus grandifolia*	*Tsuga canadensis*	*Quercus rubra*
NNF, Otto	1	N35 02.10W83 28.82	1332		*	ND		ND	*		*	ND
NNF, Otto	2	N35 01.89W83 30.29	1134	*	ND			*	ND		*	ND
NNF, Otto	3	N35 04.13W83 31.30	1073	ND	*	ND		*	*	ND		
NNF, Otto	4	N35 01.87W83 28.90	1348	ND	*	ND	*		*	ND		ND
GSMNP, NC	5	N35 35.19W83 21.49	849		ND	ND	*		ND	ND	*	ND
GSMNP, TN	6	N35 45.23W83 12.10	750		*	ND	*	ND	*	ND		ND
GSMNP, TN	7	N35 45.54W83 12.52	660	*		ND	ND		*	ND		ND
GSMNP, TN	8	N35 46.54W83 12.98	551	*	ND	ND	ND	*	ND			ND
GSMNP, TN	9	N35 42.61W83 22.86	509	ND	*	ND			*	ND		ND
GSMNP, TN	10	N35 42.82W83 23.06	477	*		ND	*		ND		*	ND
NNF, Highlands	11	N35 01.53W83 10.46	958	ND	ND		*	*	ND	ND	*	
NNF, Highlands	12	N35 02.31 W83 08.47	933	*	ND		*		ND	ND	*	ND
NNF, Highlands	13	N35 06.23W83 12.49	1321		*	ND	ND	*	ND	ND		ND
NNF, Highlands	14	N35 04.45W83 15.59	1031	*	ND	ND		*	ND	ND		ND

Most sites were in the Great Smoky Mountains National Park (GSMNP), North Carolina and Tennessee. Other sites were in the Nantahala National Forest (NNF) either west of Otto, NC or around Highlands, NC. Soil samples were collected from around all the tree species except those with the abbreviation ND, no data. Asterisks denote the focal tree species selected for the plant-soil biota interactions experiment. Each of the three inhibitor and facilitator species (in bold) were selected six times from among the pool of sites.

**Figure 2:**
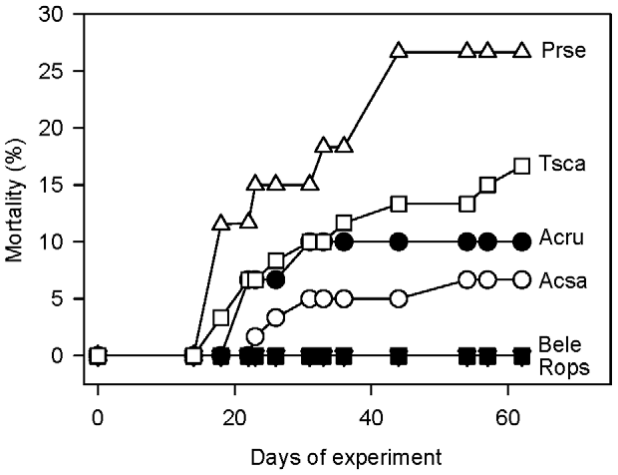
Mean seedling mortality (%) during the 62 day experiment. Species abbreviations are defined in Table 1.

Across all inocula sources, seedling mortality varied among species (GLMM, F_5,354_ = 2.36, *P* = 0.04) suggesting species have varying susceptibilities to soil-borne diseases (Fig. 2). However, there was not greater susceptibility (i.e. greater mortality) of species classified as inhibitors compared to facilitators (H3)(Fig. 2).

## Discussion

Our study used soil inocula from 14 regional sites and incorporated large amounts of regional variability. Soil biotic effects were observed infrequently, did not suggest cases of negative distance-dependent effects (H1), and did not vary consistently among *a priori* groupings of species by recruitment patterns in the field (i.e. *Inhibition* classes)(H2). We did observe some variation in the expression of disease symptoms among species of seedlings inoculated with soil from different origins. However, we did not observe that soil from conspecifics had generally more negative effects on seedling growth and survival than soil from heterospecific trees (H1). Further, inhibitor species were not more affected by soil-borne disease than species classified as facilitators (H3). Others have also shown the soil biota associated with conspecifics is not necessarily more harmful than soil biota from heterospecifics [20] in contrast with the prediction that negative distance-dependent effects predominate.

To the extent that chemical and biotic effects can be decoupled, this experiment attempted to quantify the net effect of soil biota from different sources (home vs. away) on tree seedling growth and survival. Although we failed to find empirical support for most of our predictions, we assume that differences between soil sources are driven largely by biotic interactions. Nutrient effects were considered minor because of the small relative amount of soil inocula added relative to the total volume of soil per pot and the addition of fertilizer helped to equalize any inherent variability in soil nutrients. We suspect the variability in soil nutrients among sites is greater than variability among species at a site; however, some evidence suggests that tree-soil feedbacks for some deciduous species may be driven by chemical effects [21]. A limit of our experimental design is that it provides only an estimate of the net soil biota effect of soil from different sources, and we cannot attribute effects to specific soil biota. Thus, detection of soil-borne pathogen effects (i.e. negative soil biota effects) may be obscured by the counteracting positive effects of mutualistic soil biota, like mycorrhizal fungi. However, we believe the short duration of the experiment and fertilization of pots [22] will have diminished the importance of mycorrhizal fungi since plants were unlikely to have been nutrient stressed. Since effects of mutualistic soil biota may be equally or more important in the field than in our experiment [23], we conclude that soil-borne pathogens are unlikely a driver of the recruitment patterns of the trees in the focal region.

Results from a related field experiment that transplanted seedlings near conspecific vs. heterospecific trees, at a subset of the sites used in this experiment, also did not correspond with our predictions [19]. This is not too surprising since the region experienced a drought during the time of the experiment which would have created a strong abiotic filter and potentially eliminated soil-borne disease, which are often positively correlated with soil moisture [24]. The regional drought may have also affected the soil biota; however, the growth chamber experiment revealed clear cases of damping-off disease for both *Prunus serotina* and *Tsuga canadensis* and numerous nodules were observed on the roots of *Robinia pseudoacacia*.

Theory predicts and empirical evidence often supports the idea that negative effects of soil-borne pathogens should accumulate over time and affect recruitment and help maintain species coexistence. *Prunus serotina* and *Tsuga canadensis* displayed symptoms of disease, like damping-off, illustrating their general susceptibility to soil-borne diseases (Fig. 2). These two species are known for being negatively affected by soil-borne pathogens [8], [25]. Several studies on *P. serotina* in other portions of its native range indicate greater pathogenic activity of soil associated with conspecifics than heterospecifics [3], [8] and pathogenic effects decrease with distance away from *Prunus serotina* trees [9]. However, these effects are not always observed and variation among sites, experiments (i.e. laboratory vs. field experiments), and even among individual trees have been reported [8], [9]. Here disease symptoms were associated with all soil inocula and not just inocula associated with conspecific samples. The expression of disease with soil from heterospecific trees suggest the pathogens are broadly distributed and not specifically tracking a host species. The pathogens known to cause damping-off disease (e.g. *Pythium*) can cause disease among many but not all potential host species [14] and are often described as having intermediate host-specificity. Additional research by the authors also suggests that *P. serotina* is more susceptible to damping-off disease caused by *Pythium* spp. than *A. rubrum* [15]. The general absence of mortality and expression of disease symptoms in other temperate tree species indicates variation in susceptibility among species (Fig. 2) which has been reported in studies on tropical tree species [14], [26].

We are aware of two other studies that have attempted to group tree species and compare their plant-soil biota interactions. One, a study with tropical species reported that estimates of shade tolerance were negatively correlated with susceptibility to soil microbial treatments [6]. This has important implications for understanding forest composition and successional dynamics. Similarly, another study revealed greater pathogenic activity in understory environments than gaps and hypothesized that colonization specialists were faster growing but more susceptible to seedling diseases than slower growing shade tolerant species [27]. There is also evidence of greater pathogenic activity on a temperate tree species in understory than gap environments [2]. Here we did not explicitly describe shade tolerance; however, species classified as facilitators in our study tend to also be described as shade tolerant late-successional species [28].

The other study on temperate species looked at seed disease (fungicide vs. no fungicide treatments) associated with different habitat types (gap vs. understory) and relative shade tolerance and successional status (e.g. shade intolerant vs. tolerant) of three congeneric pairs of tree species [16]. They predicted that understory soils would have greater pathogenic activity than gaps and plants described as shade intolerant (or early seral) would be most susceptible to disease. The congener comparison revealed only one of three pairs provided evidence that the shade intolerant/early seral species (*Acer negundo*) was more susceptible to disease than the shade tolerant/late seral congener (*A. saccharum*). The *Inhibition* classes used here approximate traditional successional classifications although based purely on recruitment patterns. For example, many of the facilitator species (e.g. *Acer saccharum* and *Tsuga canadensis*) in our study are typically classified as late-successional species [28]. Results from a related field experiment suggest that early seral/inhibitor species tended to experience less damage by herbivores and pathogens than later seral/facilitator species [19]. Others have also found that successional status does not necessarily correspond with distinctions in enemy impact [16] but see [2], [6], [15].

Our study reveals that the most species diverse temperate forests in the United States [29] do not appear to conform to predictions regarding soil feedback dynamics, susceptibility, and enemy impacts as they relate to the Janzen-Connell Hypothesis and related hypotheses. Our study, which used broad regional sampling and was not designed to test for many species-level effects per site, identified several responses that contradicted our predictions. The relatively short duration of the experiment may have prevented detection of negative soil feedback effects; however, other studies have detected soil borne diseases during similar and even shorter durations [3], [8], [15], [30]. Other sources of non-competitive distance or density-dependent sources of mortality (e.g. small mammals, slugs, etc.) may be structuring these temperate forests and driving the recruitment patterns previously described [19]. Alternatively, competitive distance-dependent effects (i.e. intraspecific competition for available resources and niche partitioning) may be driving recruitment patterns in the field [31] but see [32]. Overall, feedback processes appeared relatively scarce, which may be a consequence of their spatial and temporal heterogeneity in and among sites.

The coupling of the findings reported here and a related study [19] suggest that trade-offs exist causing certain species to be affected by different abiotic and biotic factors. If a component of regional species diversity is also variable interactions with biota above- and belowground then it may be extremely challenging for empirical studies to attempt to generalize across species in a trophic level. This may be especially true for studies that focus on a subset of biotic interactions (e.g. soil-borne pathogen effects) while ignoring others and use experimental designs that do not account for species-level effects. In deciduous forests of Indiana, we have documented considerable spatial variation in soil-borne pathogens around and among trees of *P. serotina* [9]. If such spatial variability is common in other systems then this may limit the prospects of identifying plant-soil biota interactions that drive recruitment patterns across multiple species occurring broadly in a region. Our study and others [16] appear to suggest temperate forests differ from their tropical counterparts [1], [6] and lack general (local to regional) patterns in plant-soil biotic interactions that structure forest communities.

## Materials and Methods

### Ethics Statement

A soil collection permit was attained to collect soil in the Smoky Mountains National Park. The remainder of the soil was collected from public property maintained by the U.S. National Forest Service which does not require a specific permit. The experiments did not involve endangered or protected species.

### Site Selection and Soil Sampling

We experimentally tested an equal number of species that had high and low probabilities of recruiting near conspecifics based on data from the US Forest Service Forest Inventory and Analysis Database (FIADB) (henceforth referred to as facilitator and inhibitor species, respectively) based on a previous study [19]. We located 14 sites in the southern Appalachians in eastern Tennessee and western North Carolina containing two or more facilitators (mean = 2.29 per site) and inhibitors (mean = 2.57) to balance our sampling design and provide landscape-level replication. Descriptions of the sites and sampling design are described in more detail in Table 2. We selected relatively flat sites which were ca. 1 ha in size and generally surrounded by sloping topography. After an initial survey of the sites, we selected the facilitator (*Acer rubrum*, *Acer saccharum*, and *Tsuga canadensis*) and inhibitor species (*Betula lenta*, *Prunus serotina*, and *Robinia pseudoacacia*) that were most common across all sites. Although two to three representative facilitator and inhibitor tree species were identified per site (average of 5.0 total species per site), the variability among sites in the region caused the identities of the focal species to vary (Table 2).

**Table 2:** Effect of soil source (conspecific vs. heterospecific) on the total mass of seedlings.

recruitment classification	species	F	df	P
inhibitor	*Betula lenta*	1.91	1,53	0.17
	*Prunus serotina*	3.32	1,53	**0.074**
	*Robinia pseudoacacia*	1.41	1,10	0.26
facilitator	*Tsuga canadensis*	3.20	1,58	**0.079**
	*Acer rubrum*	1.09	1,53	0.30
	*Acer saccharum*	0.22	1,58	0.64

Data shown in Fig. 1. P-values bold if *P*<0.10.

To establish a controlled growth chamber experiment, we collected soil from around 4–6 trees per site with approximately half being facilitator and inhibitor species per site (see Table 2 for details). Individual facilitator and inhibitor trees were haphazardly selected at each site and approximately 1 L of soil cores per tree were collected from around each focal tree species at each site during June 2007 (Table 2). We collected soil from ca. 1.5–2 m around the circumference of a tree using a sterilized soil probe sampling from 0–15 cm in depth. All cores per tree were aggregated to a single composite sample per tree. Each composite sample was homogenized and air dried for 1 week and then stored at 4°C.

### Soil Source Experiment

We performed a growth chamber experiment to quantify the effect of soil source (home vs. away soil incoula) on the growth and survival of seedlings of facilitator (*Acer rubrum*, *Acer saccharum*, and *Tsuga canadensis*) and inhibitor (*Betula lenta*, *Prunus serotina*, and *Robinia pseudoacacia*) species. These species were selected because they were frequently encountered across sites, and we had successfully germinated seeds of these species. For each focal species, the soil samples for six sites were selected at random from among the pool of soil samples from 14 sites (see “*” in Table 2) and used to inoculate pots for the experiment. Two to three focal species were selected per site with roughly half being represented by species classified as either facilitator or inhibitor tree species. Seedlings of each species were planted into 60 pots inoculated with soil from each of six sites with ten inoculations per site.

All seedlings used in the experiment were from seed purchased from commercial seed sources (Sheffield’s Seed Co., Inc., Locke, NY, USA and F. W. Schumacher Co., Inc. Sandwich, MA, USA). Seed of most species originated from Pennsylvania (*A. rubrum*, *B. lenta*, *P. serotina*, and *T. canadensis*) while others were from either Kentucky (*R. peudoacacia*) or the northern U.S. (*A. saccharum*). The pots were 2/3 filled with a mix of sterilized (once autoclaved) potting soil and sand (1∶1), then inoculated with 20 g of field soil (ca. 30 mL by volume), and then topped off with more sterile soil and sand (153 mL conetainers). Soil inocula were interpreted to primarily have biotic effects because chemical differences were likely ameliorated by the small relative amounts of soil inocula per pot (∼16% of total soil volume per pot), addition of fertilizer, and general similarity of soils at individual sites. The inocula was used to “seed” pots with soil biota representative of those found in association with different tree species and sites. Two main groups of soil inocula (or soil sources) were used which we refer to as conspecific vs. heterospecific sources [8]. Five of the 10 pots were inoculated with field soil associated with the conspecific. Soil inocula for conspecific treatments originated from one tree per site because often only one representative tree per species was identified per site. The remaining five were inoculated with heterospecific inoculum (Table 1). The inocula of the heterospecific treatments varied among sites and depended upon the species pool for each site. For example, if the focal species was a facilitator then two of the five heterospecific pots were inoculated with soil associated with two facilitator species, and the remaining three were inoculated with soil from inhibitor species and vice versa if the focal species was an inhibitor. All inocula types are for individual species and were not binned. Some sites had fewer focal species which prevented having a unique species for each heterospecific sample. To compensate, resampling of inocula of species already sampled was necessary to inoculate all five pots and maintain a balance between facilitator and inhibitor designations for the heterospecific treatment. Selections were made to optimize balancing the sampling between facilitator and inhibitor species and selection of inocula from a diverse pool of heterospecific species. For two sites with insufficient numbers of species for use as heterospecifics, we included soil inocula collected near *Quercus rubra*, a species intermediate in its recruitment classification (Table 2).

After inoculating and filling all the pots, they were watered and the next day planted with a seedling of the focal species and placed in the growth chambers. Seedlings that died up to two weeks after the start of the experiment were noted and replanted. The *Betula* seedlings experienced extremely high mortality in the first week following planting and were replanted. After the second planting, the entire rack of *Betula* seedlings was placed in a large clear plastic bag to reduce moisture stress until the seedlings were sufficiently large. The entire experiment was kept in three growth chambers which maintained constant temperature (21°C) and 12 hr light per day (PAR, ∼180 µmol×m^−2^×sec^−1^). Humidifiers were added to increase humidity in the chambers. Pots were watered based on the maximum depletion rate of a subset of pots per species to avoid over watering and leaching of nutrients while keeping plants from reaching their wilting points. Generally, larger and faster growing species were provided with more water (and fertilizer) per watering and watered at more frequent intervals to maintain sufficient soil moisture. The species were all fertilized with ½ strength Hoagland’s solution starting the fourth week post planting. They were then fertilized once per week and given an amount corresponding with their depletion rate (described above) which ranged from 8 to 29 mL. All the pots for a species were kept in one or more racks depending on their sizes and kept in the same growth chamber. Pot arrangements were re-randomized weekly and species were rotated into different chambers at the same interval. Two or more species were kept in a growth chamber and species combinations were also randomized to remove effects of chamber and neighbors. Plantings were started at staggered intervals because of asynchronous germination of multiple species with varying dormancy requirements (from approximately 7 to 100 days per species). All species did overlap for at least a portion of the experiment which lasted 62 days per species. The duration of the experiment was sufficient to observe growth in the slow growing species (e.g. *Tsuga*) without causing the faster growing species (e.g. *Robinia*) to become stressed by exceeding the capacity of the pot. Effects of soil-borne pathogens have been detected after 25 and 49 days [15], [30]. Seedling mortality was documented twice weekly. At 62 days, the plants were harvested, sorted into roots and shoots, dried, and weighed.

Plants showed no signs of nutrient stress. Soil microbes had two obvious effects on the tree seedlings. Two species (*Prunus serotina* and *Tsuga canadensis*) experienced damping-off disease symptoms typical of mortality caused by several soil-borne pathogen genera (e.g. *Phytophthora*, *Pythium*, *Rhizoctonia*, etc.) and roots of *Robinia pseudoacacia* were well nodulated. We did not assess or otherwise quantify colonization by mycorrhizal fungi.

### Analyses

Overall, the design represents an incomplete block design which prevented testing for overall effects of facilitator vs. inhibitor recruitment classification with species as a factor. This was because very few sites contained the same group of focal species (Table 2) and only a subset of the species per site were selected as focal species for the experiment. We tested the effect of inoculum source (conspecific vs. heterospecific) and site on seedling biomass (H1) to determine if soil inocula from conspecifics had negative effects relative to inocula from heterospecifics by performing separate ANOVAs for individual species using Proc MIXED in SAS version 9.13 (SAS Institute Inc., Cary NC, USA) (n = 5 per site with 6 sites per species). Degrees of freedom were estimated using the KenwardRoger option in SAS [33]. Inoculum source was treated as a fixed effect and site and inoculum×site were treated as random effects. In addition, we tested the effect of inoculum source on seedling survival (H1) with a generalized linear mixed model (GLMM; Proc GLIMMIX) in SAS with a binomial distribution of error terms (0/1 =  alive/dead; link function: logit). Individual tests were performed for each species. Inoculum source (conspecific vs. heterospecific) was the independent variable. Site was included as a random effect.

Qualitative comparisons were used to differentiate whether species classified as inhibitors were more negatively affected by soil inocula from conspecifics vs. heterospecifics relative to species classified as facilitators (H2). We relaxed the typical significance threshold of *P*≤0.05 to *P*<0.10 to test H1–2 because our study incorporates considerable regional variability in both biotic (e.g. composition of heterospecific species across sites, variation in microbial communities among sites, etc.) and abiotic factors (e.g. variation in soil characteristics among sites) that increase the probability of committing a Type II statistical error. We compared the susceptibility of species and species groups (H3) with a GLMM (Proc GLIMMIX; binomial distribution of error terms; link function: logit). Seedling survival was the response variable. Species was treated as a fixed effect.
